# Depression prevalence and primary care among vulnerable patients at a free outpatient clinic in Paris, France, in 2010: results of a cross-sectional survey

**DOI:** 10.1186/1471-2296-14-151

**Published:** 2013-10-11

**Authors:** Claire Rondet, Philippe Cornet, Bacha Kaoutar, Jacques Lebas, Pierre Chauvin

**Affiliations:** 1 INSERM, U707, Research Group on the Social Determinants of Health and Health Care, Paris F-75012, France; 2 UPMC Univ Paris 06, School of Medicine, Department of General Practice, Paris F-75012, France; 3 AP-HP, Hôpital Saint-Antoine, Polyclinique Baudelaire, Paris F-75012, France

**Keywords:** Depression, Primary care, Seeking healthcare, Homeless, Immigrants, Paris, France

## Abstract

**Background:**

Data on the prevalence of depression and on how a depressive episode prompts the sufferer to seek primary care are not scarce, but the available evidence on the prevalence of depression among immigrants and poor people who frequent general practice facilities is scarce. The Baudelaire Outpatient Clinic at the Saint-Antoine Hospital in Paris provides free medical and social assistance to the poor and/or uninsured. The goal of our study was to estimate the prevalence of depression among these outpatients, to characterize this depressed population, and to analyze its demand for primary care for depressive episodes.

**Methods:**

From September to December 2010, we conducted a cross-sectional, observational survey among users of the Baudelaire Outpatient Clinic. French-speaking patients attending the clinic between September 15 and December 30, 2010 who agreed to answer a questionnaire administered face-to-face before their consultation were included in the study. The chi-squared test (or Fisher’s exact test for small samples) was used for the comparisons of proportions. Logistic regression models were estimated, along with the odds ratios (OR) and their 95% confidence intervals (95% CIs), for the multivariate analysis of factors associated with depression and healthcare-seeking. Models were estimated separately for men and women, since sex was an interaction factor. The statistical analyses were performed using Stata v. 10 software (StataCorp LP, College Station, Texas, USA).

**Results:**

Of the 250 patients included (mean age: 45 years), 52.0% were men and 52.4% were immigrants. Close to 40% of them reported having no supplemental health insurance. The estimated prevalence of depression in this population was 56.7%. Depression was more prevalent among the women, immigrants, and people from the poorer socioeconomic groups. Only half of these depressed patients, mostly women, reported having discussed their depression with a physician. French nationality and complete health insurance coverage were associated with more-frequent healthcare-seeking. Few patients reported having been asked about their morale by the physician they consulted, and almost 80% would have liked to be asked about this more often.

**Conclusion:**

Depression is a real public health problem, particularly among people from disadvantaged backgrounds, and should be included in their overall management.

## Background

A real public health problem in France, depression is common, often has serious consequences and is frequently accompanied by difficulties in social integration. The prevalence of depression in France is estimated at 5 to 12% in the general population [1-3] and increases markedly in the poorest populations [4,5], reaching 41% among the least well-off women. The characteristics associated with depression have been described extensively in the general population [1,2] but little among the more vulnerable [4,6]. Not much is known about healthcare-seeking during a depressive episode [7-10], particularly among the poorest groups [11]. The Baudelaire Outpatient Clinic at the Saint-Antoine Hospital in Paris is a general medicine outpatient clinic in one of 32 university-affiliated general hospitals. It is frequented by vulnerable individuals because it offers free access to medical and social assistance for the poor and/or uninsured. Patients have access to general medical care, nursing care, social assistance, additional medical examinations, and, if need be, the hospital’s specialty departments. Our goals were to estimate the prevalence of depressive episodes in this vulnerable population seeking primary care, to define the sociodemographic characteristics of the depressed patients, and to assess their demand for healthcare.

## Methods

Data were collected in two steps. First, a qualitative study using semistructured interviews was carried out to record the users’ perceptions, attitudes and experiences regarding depression. The aim was to maximize the collecting of data from a diverse sample of these subjects. Data were collected from June to mid-August 2010 in the Paris metropolitan area from 30 people (17 women and 13 men) selected at random and approached at general medical consultations (n=13), at child and maternal centers (n=9), or on public transport (n=8), and they were interviewed face-to-face using an interview guide (see Additional file 1). The interviews were conducted until saturation. Notes were taken during the interview and were later analyzed in terms of general perceptions of depression, any personal history of depression, and healthcare-seeking during depressive episodes. The responses were subjected to a qualitative content analysis. Two raters (CR and PC) independently read and categorized the responses, then met and conferred until they agreed on a common classification. Using this analysis, we devised a quantitative questionnaire on these and other dimensions.

Second, an anonymous, observational, cross-sectional survey was conducted in a sample of patients attending general medical consultations at the Baudelaire Outpatient Clinic at the Saint-Antoine Hospital in Paris. One day a week, all French-speaking adult patients attending a consultation were asked, after they had given proper informed consent, to take part in this study. Excluded from the study were non-French speakers, individuals with behavioral disorders incompatible with the administration of a questionnaire (e.g., an acute psychiatric disorder or alcoholism) and/or requiring urgent care.

### Survey population and outcome

A total of 250 French-speaking patients who attended the clinic between September 15, 2010 and December 30, 2010 agreed to answer a questionnaire administered face-to-face before their consultation and were included in the study. A total of 5 patients were excluded (3 refused to participate, 1 had a psychiatric disorder and 1 required urgent care). This survey did not fall into the category of biomedical research (as defined by French law) and did not involve collecting any personal identification data. Therefore, it did not require ethical approval.

The questionnaire contained 62 questions (most requiring closed-ended responses) designed to gather sociodemographic data and included the Mini-International Neuropsychiatric Interview (M.I.N.I.) [12], which contains questions about major depressive episode the type of healthcare sought, and the reasons for not seeking healthcare during depressive episodes. For major depressive episode, the M.I.N.I. [12] uses a set of ten questions to identify depressive symptoms during the two weeks preceding the interview. The responses were used to calculate an individual score which, with appropriate sensitivity and specificity, was used to identify the individuals with depressive symptoms.

### Independent variables

As regards the respondents’ sociodemographic status, we considered their age (four categories), their self-declared nationality (two categories: French and foreign immigrants), their sex, their civil status (four categories: single, married or living with partner, divorced, and widow/widower).

As for their socioeconomic status, we considered their educational level (in two categories: high school and postsecondary), their occupational category (initially using the 8-category INSEE nomenclature: farmers, tradespeople and shopkeepers, managerial staff, intermediary occupation, manual staff, clerical staff, retired and unemployed people. However, for statistical reasons, we used two categories: managerial/professional staff and other), their health insurance status (in four categories: standard social security, universal [which is free healthcare for low-income individuals], government medical assistance, and none).

The respondents were also asked whether or not they had a regular physician and about their perceived social isolation.

### Analysis of healthcare-seeking

To analyze healthcare-seeking, the interviewees were asked whether they had talked to a physician about their depression (past or current episode). If the response was negative, a series of closed-ended questions was used to explore why. Lastly, the physician’s involvement in terms of psychological health was investigated by means of a general question (During your visits, has a physician shown an interest in your morale, mental state, or mental well-being?). Lastly, we asked the interviewees if they would like the physician to ask them about this more often.

### Statistical methods

The chi-squared test (or Fisher’s exact test for small samples) was used for the comparisons of proportions. Logistic regression models were estimated, along with the odds ratio (OR) and their 95% confidence intervals (95% CIs), for the multivariate analysis of factors associated with depression and healthcare-seeking. Models were estimated separately for men and women, since sex was an interaction factor. The statistical analyses were performed using Stata v. 10 software (StataCorp LP, College Station, Texas, USA).

## Results

During the 4-month survey period, 255 individuals sought healthcare. Among this eligible population, 2 were excluded because of a behavior or medical problem, and 3 refused to participate (3 men, 2 foreign immigrants and 1 French national; refusal rate=2%). Therefore, the survey population consisted of the 250 individuals who were interviewed.

### Sociodemographic characteristics

The study participants (mean age: 45 years; 52.0% men) were generally of low socioeconomic status, 52.4% were immigrants, and 31.2% reported having a postsecondary education. Only 40.0% had a job on the day of the survey, 9.6% had managerial/professional positions, 10.0% were unemployed, and 16.0% were retirees (Table 1). Half of the interviewees (55.2%) had the standard health insurance enjoyed by most of the population in France (i.e., basic insurance with the social security system together with private supplemental insurance), 20.0% had a health insurance plan intended for the poor (CMU, or universal health insurance), 18.8% were insured through a specific mechanism for undocumented immigrants (AME, or public medical assistance), and 6.0% were uninsured. More than a third (37.6%) had no supplemental insurance (and were therefore only partially covered, by the basic social security insurance).

**Table 1:** Characteristics of the survey population (total and by sex) and of the depressed population (by sex)

	**Total population**		**Male population**					**Female population**				
			**Total**		**Depressed**			**Total**		**Depressed**		
	**N**	**%**	**n**	**%**	**N**	**%**	p*	**n**	**%**	**n**	**%**	p*
**Age (years)**												
Under 25	20	8.0	10	50.0	5	50.0	0.74	10	50.0	7	70.0	0.11
25 to 44	118	47.2	68	57.6	35	51.5		50	42.3	32	64.0	
45 to 59	62	24.8	27	43.5	17	63.0		35	56.4	25	71.4	
Over 60	50	20.0	24	48.0	12	50.0		26	52.0	11	42.3	
**Nationality**												
French	118	47.2	51	43.2	24	47.0	0.24	67	56.8	32	47.8	<0.001
Foreign	132	52.8	78	59.1	45	57.7		54	40.9	43	79.6	
**Occupational category**												
Managerial/professional	24	9.6	8	33.3	1	12.5	0.015	16	66.6	4	25.0	0.001
Other	225	90.4	120	53.3	68	56.7		105	46.7	71	67.6	
**Civil status**												
Single	108	43.2	62	57.4	37	59.7	0.12	46	42.6	32	69.6	0.13
Married or living with partner	109	43.6	55	50.5	26	47.3		54	49.5	28	51.9	
Divorced	22	8.8	9	40.9	3	33.3		13	59.1	8	61.5	
Widow/widower	11	4.4	3	27.3	3	100.0		8	72.7	7	87.5	
**Health insurance**												
Standard	138	55.2	60	43.5	24	40.0	0.035	78	56.5	39	50.0	0.002
Universal	50	20.0	30	60.0	21	70.0		20	40.0	15	75.0	
Public medical assistance	47	18.8	31	65.9	20	64.5		16	34.1	14	87.5	
None	15	6.0	8	53.3	4	50.0		7	46.7	7	100.0	
**Living arrangement**												
Family (spouse, children, other)	145	58.0	63	43.4	30	47.6	0.35	82	56.6	47	57.3	0.30
Non-relative	24	9.6	16	66.6	8	50.0		8	33.3	6	75.0	
Living alone	80	32.0	49	61.2	30	61.2		31	38.8	22	71.0	
**Treating physician**												
Yes	205	82.0	93	45.4	50	53.8	0.63	112	54.6	69	61.6	0.76
No	45	18.0	36	80.0	19	52.8		9	20.0	6	66.7	
**Educational level**												
High school	172	68.8	95	55.2	55	57.9	0.09	77	44.8	51	66.2	0.20
Postsecondary	78	31.2	34	43.6	14	41.2		44	56.4	24	54.5	
**Feeling of isolation**												
Very alone	28	11.2	18	64.3	16	88.9	<0.001	10	35.7	9	90.0	0.12
Rather alone	84	33.6	49	58.3	28	57.1		35	41.7	23	65.7	
Supported	108	43.2	46	42.6	19	41.3		62	57.4	37	59.7	
Strongly supported	30	12.0	16	53.3	6	37.5		14	46.7	6	42.9	

*Comparison with the distribution for the total population of the same sex.

Nearly half (43.2%) of the interviewees reported that they were married or living with someone, and 43.6% were single. Only 32.0% of the interviewees reported that they were living alone, 58.0% said that they were living with a family member, and nearly 9.6% were living with a non-relative. Lastly, 82.0% reported having a regular physician (even if he or she was not always officially registered with the appropriate Department of Health office).

### Prevalence of depressive episodes and associated characteristics

The M.I.N.I. indicated an estimated prevalence of major depressive episodes of 56.7% among the people who were attending the Baudelaire Outpatient Clinic (95% CI=[51.4%-63.8%]). The prevalence was 53.5% (95% CI=[44.8%-62.2%]) in men and 62% (95% CI=[53.2%-70.8%] in women (*p*=0.17).

In univariate analysis, in men and women alike, not having a managerial/professional position and/or the standard health insurance was significantly associated with a risk of depression (Table 1): 56.7% of the men who were not in a managerial/professional position were depressed (*p*=0.02) versus 67.6% of the women (*p*<0.001). A majority (70.0%) of the men who had health insurance for the poor and 64.5% of those who were receiving AME were depressed versus 40.0% of the men with the standard health insurance (*p*=0.04). All the uninsured women were depressed (as were 87.5% of those receiving AME and 75.0% of those with CMU) versus 50.0% of the women with the standard health insurance.

After adjustment for age, the characteristics associated with a risk of depression in univariate analysis were female gender, not being in a managerial/professional position (*p*<0.001), a low educational level (*p*=0.05), being single (*p*=0.03), not having the standard health insurance (*p*<0.05) and foreign nationality (*p*=0.002).

Multivariate analysis in women (Table 2) showed that not being in a managerial/professional position was associated with a five-fold greater risk of depression (*p*=0.02). Living alone was associated with a three-fold greater risk (*p*=0.05). Although the association was not statistically significant (*p*=0.17), the women receiving AME had a five-fold greater risk of depression than those with the standard health insurance.

**Table 2:** Characteristics associated with depression in multivariate analysis, by sex (logistic regression)

	**Women**		**Men**	
	***p*-value**	**OR [95% CI]**	***p-*value**	**OR [95% CI]**
**Age (years)**				
Under 25		Ref.		Ref.
25 to 44	0.99	1.02 [0.17-6.05]	0.70	0.74 [0.16-3.47]
45 to 59	0.62	1.58 [0.26-9.00]	0.60	1.56 [0.30-8.13]
Over 60	0.39	0.45 [0.07-2.75]	0.98	0.98 [0.19-5.10]
**Health insurance**				
Standard		Ref.		Ref.
Universal	0.08	3.02 [0.87-10.50]	0.048	2.86 [1.01-8.10]
Public medical assistance	0.07	5.12 [0.90-29.20]	0.09	2.62 [0.85-8.10]
None	0.80	1.28 [0.19-8.70]	0.80	1.28 [0.19-8.70]
**Occupational category**				
Managerial/professional		Ref.		Ref.
Other	0.02	5.37 [1.30-22.20]	0.23	3.94 [0.42-37.20]
**Living arrangement**				
Family		Ref.		Ref.
Non-relative	0.36	2.46 [0.36-16.70]	0.56	0.68 [0.18-2.56]
Living alone	0.05	3.05 [0.98-9.50]	0.40	1.48 [0.60-3.70]
**Feeling of isolation**				
Strongly supported		Ref.		Ref.
Supported	0.71	1.30 [0.30-5.20]	0.68	1.32 [0.40-4.90]
Rather alone	0.90	0.90 [0.20-4.40]	0.53	1.55 [0.40-6.00]
Very alone	0.60	2.13 [0.13-34.40]	0.02	9.90 [1.37-71.50]

Multivariate analysis in men (Table 2) showed that the risk of depression was three-fold higher in those with health insurance for the poor (*p*=0.05) than in those with the standard health insurance, and 10 times higher in those who reporting having a strong feeling of isolation (*p*=0.02). Unlike in women, living alone or not having a managerial/professional position did not seem to be associated with a risk of depression in men.

### Healthcare-seeking during a depressive episode

Altogether, 61.6% of the women and 53.8% of the men had a regular physician (*p*=0.01). During the interviews, of the 144 people diagnosed as depressed, only 52.8% reported having talked to a physician about their depression. This seemed to apply more often to women, although the difference was not significant (*p*=0.07, and the number was small). Only 44.3% of the foreigners had talked to a physician about their depression versus 66.1% of the French nationals (*p*=0.01). Close to 60% of the participants without supplemental health insurance had not talked to a physician about their depression versus 39.2% of those with supplemental insurance and 33.3% of those with CMU (*p*=0.02). Lastly, all the participants in a managerial/professional position had done so versus half of those in other occupational categories (*p*=0.03).

In multivariate analysis (Table 3), only supplemental health insurance and French nationality were significantly associated with seeking healthcare. The participants who had supplemental insurance were twice as likely to have consulted a physician than those who did not (*p*=0.05), and those with CMU were three times more likely (*p*=0.02). Lastly, foreigners were three times less likely to have seen a physician than the French nationals (*p*=0.01).

**Table 3:** Characteristics associated with healthcare-seeking during a depressive episode (logistic regression)

	**Model 1**^**1**^		**Model 2**^**2**^		**Model 3**^**3**^	
	**P**	**OR [95% CI]**	**p**	**OR [95% CI]**	**p**	**OR [95% CI]**
**Age (years)**						
Under 25		Ref.		Ref.		Ref.
25 to 44	0.37	1.86 [0.48-7.22]	0.40	1.78 [0.47-6.70]	0.62	1.38 [0.38-5.00]
45 to 59	0.13	2.96 [0.73-12.11]	0.14	2.82 [0.72-11.12]	0.22	2.33 [0.61-8.92]
Over 60	0.73	0.77 [0.17-3.45]	0.68	0.73 [0.17-3.22]	0.55	0.64 [0.15-2.76]
**Sex**						
Men		Ref.		Ref.		Ref.
Women	0.14	1.71 [0.85-3.48]	0.13	1.71 [0.85-3.42]	0.14	1.70 [0.85-3.42]
**Nationality**						
Foreign		Ref.		Ref.		
French	0.07	2.28 [0.93-5.63]	0.01	2.67 [1.25-5.72]		
**Health insurance**						
None		Ref.				Ref.
Private insurance	0.48	1.41 [0.54-3.63]			0.05	2.23 [1.01-4.96]
Universal	0.05	2.60 [0.98-6.88]			0.02	3.13 [1.22-7.99]

^1^The multivariate analysis included the following variables: age, sex, nationality and health insurance.

^2^The multivariate analysis included the following variables: age, sex and nationality.

^3^The multivariate analysis included the following variables: age, sex and health insurance.

## Discussion

Our study sought to determine the prevalence of major depressive episodes among vulnerable patients, to define the socioeconomic characteristics of depressed people, and to explore their healthcare-seeking. We found that 56.7% of this population suffered from depression. The characteristics associated with depression were, as in the general population, female gender, being a foreigner, living alone and having a low socioeconomic status. Only half of the depressed patients had talked to a healthcare professional about their depression. Almost two-thirds of them indicated that, during their medical consultations, the physician did not show an interest in their morale, mental state, or mental well-being. However, almost 80.0% of the interviewees reported that they would like the physician to ask them about this more often.

We estimate the prevalence of depression among users of the Baudelaire Outpatient Clinic to be 57%, which is markedly higher than in surveys among the general population. This high prevalence is comparable to the results of the only similar French survey found in the literature, which was conducted among patients at a similar outpatient clinic 10 years ago in the southern city of Avignon. The authors estimated the prevalence of psychiatric disorders at 27% and indicated a prevalence of 12.0% for major depressive disorder and 21.0% for anxiety disorder [13].

The high prevalence of depression observed in our patient population can also be compared with the estimated prevalence reported in recent French studies of two specific socially disadvantaged and marginalized populations. The SAMENTA study, which was carried out in 2009 in a representative sample of homeless people in the Paris metropolitan area [4], reported an estimated prevalence of depressive episodes of 22.5%. In a study conducted between September 2003 and July 2004 in men in three types of penitentiaries in France (prisons, central prisons, and detention centers), Fallissard et al. [6] found that the depression prevalence varied from 17.9% to 23.0%, depending on whether the diagnosis was made by a clinician or using the M.I.N.I. Lastly, in 2000, in a study conducted at 12 public centers for free medical check-ups, Royer et al. [5] examined the link between precarious situations and anxiety/depressive disorders. They found a mean prevalence of 27.2% in women and 16.9% in men, but these figures increased respectively to 41.7% and 34.4% among the most disadvantaged individuals.

While it is clear that our study population was not comparable to the general population, we wanted to underscore the fact that our estimated prevalence of depression is considerably higher than that in the general population. In fact, these prevalence rates ranged from 5.0% [2] to 11.7% [14,15]. Our study found that vulnerable patients have the same characteristics as those described in the general population but that they are affected more often.

Several factors can explain the much higher prevalence of depression among the patients in our study than in the general population. First, it was a survey among people attending a clinic, who are, by definition, less healthy than people in the general population. Furthermore, the patients in our study were interviewed in a hospital setting and at an outpatient clinic. Therefore, in addition to having major social problems, they may have had organic diseases, some of them chronic and anxiogenic [16]. This bias is therefore probably responsible for overexposure to the risk of depression. Overdiagnosis of depression was a possibility in that a diagnosis using the M.I.N.I does not take into account any underlying diseases, substance abuse, or reactional states. The M.I.N.I. is a very good screening tool (particularly in general medicine), but it has only been validated in the general population and among patients consulting physicians [12]. Nevertheless, it has been used in prisons [6] and among homeless people [4] (see below). Conversely, some biases may have led to an underestimation of the prevalence of depression. There may have been comprehension difficulties, notably among foreigners who, although French- speaking, did not all have the same level of expression and comprehension in French. As well, some patients may have been bothered by the personal nature of certain questions. Lastly, some depressed patients on appropriate treatment may have been incorrectly diagnosed as not being depressed using the M.I.N.I. (which is a strictly symptom-based tool) [12].

### Factors associated with depression

Our findings concerning the factors associated with depression are consistent with those of previous surveys in the general population [1-3,17-19]. Among other things, we found that women are at greater risk for depression, although it should be borne in mind that there may have been some underreporting bias, since men may find it more difficult to admit to states such as sadness, a loss of interest, or a lack of self-confidence [18]. In our study population, the women living alone had a three-fold greater risk of a depressive episode. Surveys in the general population have estimated that a person living alone is 1.5 to 2.4 times more likely to experience a depressive episode than someone who is married or living with a partner (for women and men alike) [3]. More generally, we found that, regardless of their relationship with their cohabitant(s), the participants who were not living alone seemed to be at lower risk for a depressive episode. Of course, in our cross-sectional survey, the significance of such an association (and its causal nature) remains open to discussion, since a depressive state may also have a harmful impact on the maintenance of a conjugal relationship. Likewise, the feeling of isolation (significantly associated with depression in multivariate analysis), may be a cause or a consequence of a depressive state.

We also found that depression was more prevalent in the individuals in the least-advantaged social and occupational categories. The SIRS survey, which was conducted in the general population in the Paris metropolitan area, the same urban context as for our study, found, in 2005, that people who were not in managerial/professional positions were at a two-fold greater risk for depression than the others [14,15]. In our study, this risk was five-fold higher in the women who were not in managerial/professional positions and four-fold higher in their male counterparts. This much greater risk might be explained by the fact that we classified the unemployed (58% of the interviewees) together with those in non-managerial/non-professional positions. This result may therefore reflect the risk of depression in individuals in non-managerial/non-professional positions, but, above all, in those who did not have a job. Indeed, the risk of depression in the participants who were unemployed was 2.7-fold higher than that in those who were employed (*p*<0.001), this for both genders. This result is indicative of the role of the rupture of social links (in this case, in the workplace) in the onset of a depressive episode. Here, too, it should be stressed that the relationship between being unemployed and depression is not one-way (severe depression can also cause one to be out of work, at least temporarily).

### Depression more prevalent in Immigrants

In our survey, as in those conducted in the general population in France [1-3,15,16], the prevalence of depression was higher in foreigners than in French nationals. Such a difference is also observed in other European countries [11,20]. This was particularly so for the women in our study, since close to 80% of the immigrant women were depressed compared to less than half of the French women (*p*<0.001). Generally speaking, most immigrants report worse perceived general [21,22] or mental [20] health status than that of people in their host country (at least after living in the host country for some time, since, for the new migrants, a health migrant effect is often reported [23]). This can be explained by certain conditions and circumstances associated with migration (violence, dangerous journeys, [24-26]), conditions in the host countries. In France, among other countries, immigrants more often have jobs with difficult working conditions, less security and lower salaries than their French counterparts for the same level of qualification [21]. The break with their country of origin, their family and their culture, and their social isolation in France (especially in a city like Paris) probably explain this greater risk as well. Indeed, most of the interviewees were immigrants, for whom it is known that acculturation is a major risk factor for depression [27-29], and, moreover, close family ties are important in preventing anxiety/depressive disorders [30].

### Only half of the participants suffering from depression had talked to a healthcare professional about it

Only half of the depressed individuals in our survey reported having talked to a physician about their depression compared to 60% of the people in the general population who had consulted a healthcare professional, as estimated by the *Baromètre santé* survey conducted in France in 2005. This difference is probably due to methodological differences: the *Baromètre santé* survey was conducted by telephone and considered healthcare seekers those people who had seen a healthcare professional (of any type) and people who were taking psychotropic medications (whether or not they were seeing a healthcare professional), whereas we only took into account medical consultations in our survey. When asked, “During your visits, has a physician shown an interest in your morale, mental state, or mental well-being?”, almost two-thirds of the interviewees answered “no”! The vast majority (78.7%) of these patients indicated that they would have liked the physician to ask them about this more often, and nearly 20% stated that they would like such questioning to be routine. Contrary to what some physicians might think, reticence in addressing these issues does not stem from the people themselves (including socially disadvantaged individuals attending a free clinic for the poor and/or uninsured). In our survey population, depressed foreigners were less likely to have talked to a physician (44% versus 66% of the depressed French nationals, *p*=0.01). This disparity was also noted in the general population in the 2005 SIRS survey, in which only 34% of the depressed foreigners had sought out a healthcare professional for depression (versus 52% of French nationals, *p*=0.03). Regardless of the reason for consultation, foreigners in France generally seek healthcare less often than French nationals, as shown in a 2005 survey by the Department of Research, Studies and Statistics (DREES) of the French Ministry of Health [31]. In other European countries as well, for example Spain and the UK, it has been shown that ethnic minorities use secondary care services less than others [32,33].

## Conclusion

In light of our results, especially the observation that over three-fourths of the patients reported that the physician did not address mental health in any way during their visits and that close to 80% of them would, in fact, have liked the physician to ask them about this more often, we would recommend that depression and mental well-being be included more systematically in the basic interview of all patients in primary care, regardless of their social background. This does not mean that their social background does not need to be taken into account. On the contrary, along with other authors who described the different barriers to care for depression in minorities and who call attention to the fact that physicians give less information about depression to these patients [34,35], we would also recommend that three components be explored with poor or underserved patients, especially if they are immigrants: the patient’s explanatory model of depression, the social and financial barriers to adherence to its treatment, and the fears and concerns about medications or their potential side effects. Of course, family physicians cannot fully overcome, through their own practice, all the social aspects and consequences of health (particularly, in this case, mental health) disparities faced by minorities, but attention should clearly be given to broader economic, sociocultural and institutional barriers to care, and it seems important for the management of depression that it be part of the patient’s overall care, particularly among the most disadvantaged”.

## Competing interests

The authors declare that they have no competing interests.

## Authors’ contributions

CR designed the study, interviewed the patients at the outpatient clinic and conducted the semidirective interviews for the qualitative survey. She performed the statistical analysis and interpreted data. She also drafted the manuscript. PC was involved in drafting the manuscript and in critically reviewing it for important intellectual content. BK and JL were involved in critically reviewing it for important intellectual content. PC made substantial contributions to the conception and design, and the data analysis and interpretation. He contributed to the drafting of the manuscript and gave final approval of the version to be published. All authors read and approved the final manuscript.

## Pre-publication history

The pre-publication history for this paper can be accessed here:

http://www.biomedcentral.com/1471-2296/14/151/prepub

## Supplementary Material

Additional file 1 Semistructured interview guide. Click here for file
