# Supplementary material for: Depression prevalence and primary care among vulnerable patients at a free outpatient clinic in Paris, France, in 2010: results of a cross-sectional survey
Source: BMC Fam Pract. 2013 Oct 11;14:151. doi: 10.1186/1471-2296-14-151 (PMC3852793; doi:10.1186/1471-2296-14-151)
Supplement: Additional file 1 — Semistructured interview guide. [file 1471-2296-14-151-S1.pdf]

Additional file 1: Semistructured interview guide

Date of interview	
Interviewer	
Interviewee's sex	
Interviewee's age	
Interviewee's occupational category	
**Theme**	**Questions**
Perception of depression	For you, what is depression?<br>How do you perceive this condition?<br>What do you think of depressed people?<br>Do you think that depression is a disease like other diseases?<br>Do you think that depression can be cured?<br>Are you afraid of depression? Why?<br>Do you think that being depressed is shameful?
About the interviewee	Have you ever had depression?<br>Did you talk about depression with the doctor?<br>Did you hesitate to talk about it with the doctor?<br>Why?
Treatment of depression	Who should you talk to in case you are depressed?<br>How could the healthcare system help depressed people?<br>Do you think medications can help in cases of depression? Why?
